# Supplementary material for: Microbial Degradation, Spectral analysis and Toxicological Assessment of Malachite Green Dye by Streptomyces exfoliatus
Source: Molecules. 2022 Sep 30;27(19):6456. doi: 10.3390/molecules27196456 (PMC9572514; doi:10.3390/molecules27196456)

Figure S1: **Constituents of edible oil waste Identified by GC-MS Analysis**

9-Octadecenoic acid (Z)-, methyl ester
Formula C19H36O2, MW 296, CAS# 112-62-9, Entry# 4747
Oleic acid, methyl ester

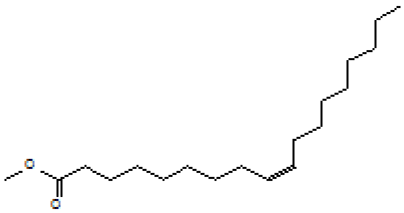

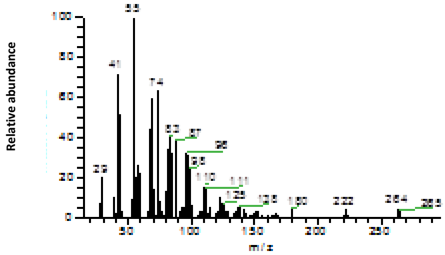

9-OCTADECENOIC ACID (Z)-, METHYL ESTER
Formula C19H36O2, MW 296, CAS# 112-62-9, Entry# 186152
9-OCTADECENOIC ACID, METHYL ESTER

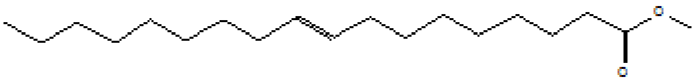

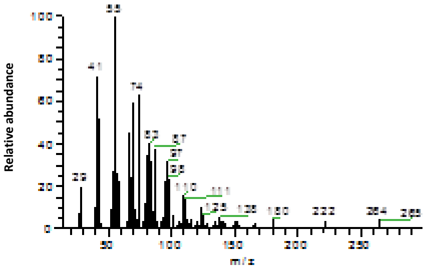

11-Octadecenoic acid, methyl ester
Formula C19H36O2, MW 296, CAS# 52380-33-3, Entry# 19082
Methyl 11-octadecenoate

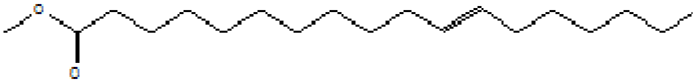

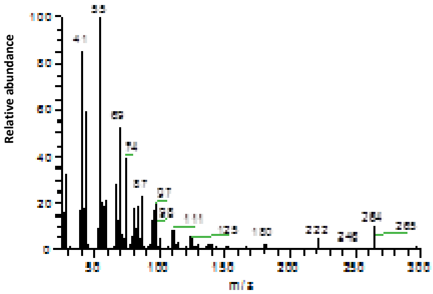

6-OCTADECENOIC ACID, METHYL ESTER, (Z)-
Formula C19H36O2, MW 296, CAS# 2777-58-4, Entry# 186134
METHYL (6Z)-6-OCTADECENOATE#

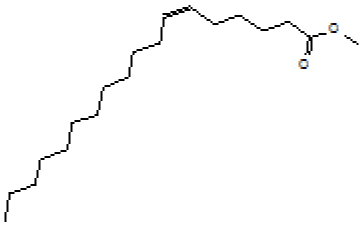

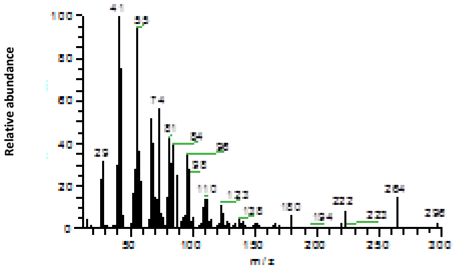

11-OCTADECENOIC ACID, METHYL ESTER
Formula C19H36O2, MW 296, CAS# 52380-33-3, Entry# 186176
OCTADEC-11-ENOIC ACID METHYL ESTER

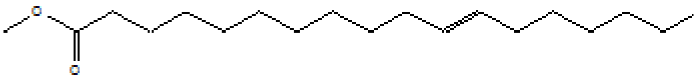

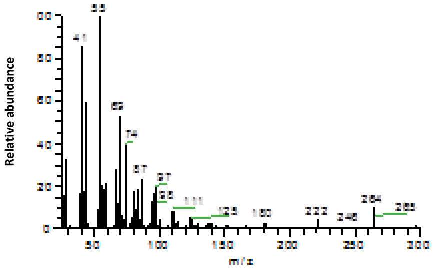

6-OCTADECENOIC ACID, METHYL ESTER, (Z)-
Formula C19H36O2, MW 296, CAS# 2777-58-4, Entry# 186134
METHYL (6Z)-6-OCTADECENOATE#

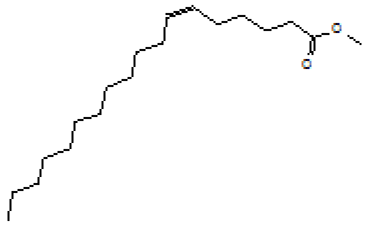

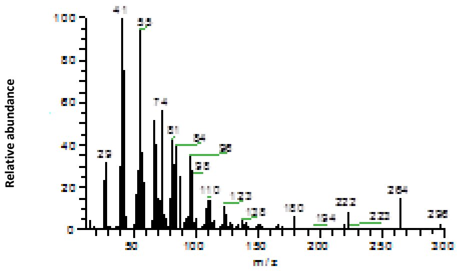

6-Octadecenoic acid, methyl ester, (Z)-
Formula C19H36O2, MW 296, CAS# 2777-58-4, Entry# 965
Methyl cis-6-octadecenoate

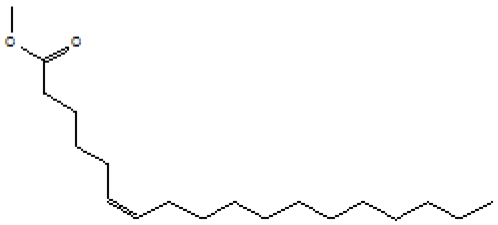

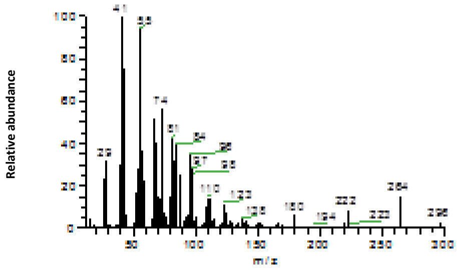

11-Octadecenoic acid, methyl ester
Formula C19H36O2, MW 296, CAS# 52380-33-3, Entry# 19082
Methyl 11-octadecenoate

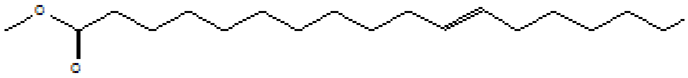

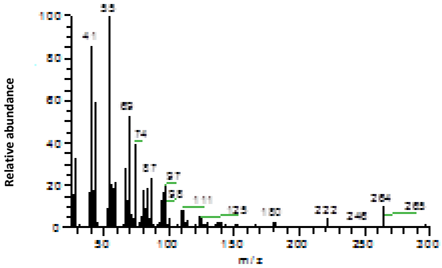

OCTADECANOIC ACID, METHYL ESTER
Formula C19H38O2, MW 298, CAS# 112-61-8, Entry# 187918
METHYL OCTADECANOATE

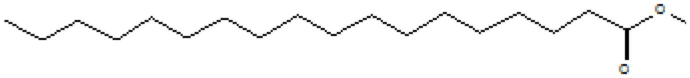

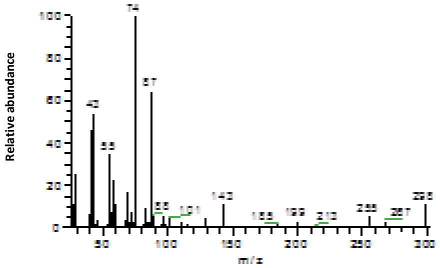

Supplement: Supplementary file 1 [file molecules-27-06456-s001.zip › molecules-1896376-supplementary.pdf]
